# Supplementary material for: Heating Drinking Water in Cold Season Improves Growth Performance via Enhancing Antioxidant Capacity and Rumen Fermentation Function of Beef Cattle
Source: Antioxidants (Basel). 2023 Jul 26;12(8):1492. doi: 10.3390/antiox12081492 (PMC10451963; doi:10.3390/antiox12081492)
Supplement: Supplementary file 1 [file antioxidants-12-01492-s001.zip › antioxidants-2494967-supplementary.pdf]

antioxidants

Supplementary material

Table S1. Diet composition and nutrition levels (%, DM basis)

Ingredient Composition	Content
Corn	35.0
DDGS	5.00
Corn germ meal	15.0
Cotton seed	4.00
Whole corn silage	34.0
Wheat straw	5.50
Premix	0.50
$NaHCO_3$	0.50
NaCl	0.50
Analyzed nutritional composition	
DM	51.2
CP	11.3
ADF	17.4
NDF	32.8
EE	4.07
Calculated nutritional composition	
NEg; Mcal/kg	1.56

DM: dry matter; CP: crude protein; NDF: neutral detergent fiber; ADF: acid detergent fiber; EE: ether extract; Premix: Fe 12 g/kg, Mn 1 g/kg, Cu 1 g/kg, Zn 11 g/kg, I 30 mg/kg, Se 30 mg/kg, Co 20 mg/kg, Vitamin A 450,000 IU/kg, Vitamin D_3 60,000 IU/kg, Vitamin E 2000 mg/kg; NEg was estimated from CNCPS (6.0) values.

Table S2. Sequences of primer used to analyze gene expression in rumen epithelial tissue by quantitative PCR.

Target	Primer Sequences (5′-3′)	Gene Base Number	PCR Product Size (bp)
β-actin	F：ACTGTTAGCTGCGTTACACCCT	22	167
	R：TGCTGTCACCTTCACCGTTC	20	
ZO-1	F：TCAACCACAATCTCTTGCCGAATG	24	125
	R：GGTCTCCACGCCACTGTCAA	20	
Claudin-4	F：CTTCCATCCTTCCTGCTCCACTCT	24	163
	R：CCTCTAAACCTGTCCGTCCACTCT	24	
Occludin	F：GCTACGGCTATGGCTACGGTTATG	24	193
	R：AGGACGGCGGTCACTATTATCACA	24	
MCT1	F：GGTGGAGGTCCTATCAGCAGTGT	23	228
	R：TGCCAGCGGTCGTCTCTTATAGAA	24	
MCT4	F：GGAGGTGGAGCAGTTCCTGAAGA	23	131
	R：ACGAGCGTTGACGGTCTCTGT	21	
SGLT1	F：CATCTCTACCGCCTGTGCTGGA	22	218
	R：GCTGCCTCCTCTTCCTTCGTCAT	23	

ZO-1, zonula occludens-1; MCT1, monocarboxylic acid transporters 1; MCT4, monocarboxylic acid transporters 4; SGLT1, sodium-dependent glucose-linked transporter-1.

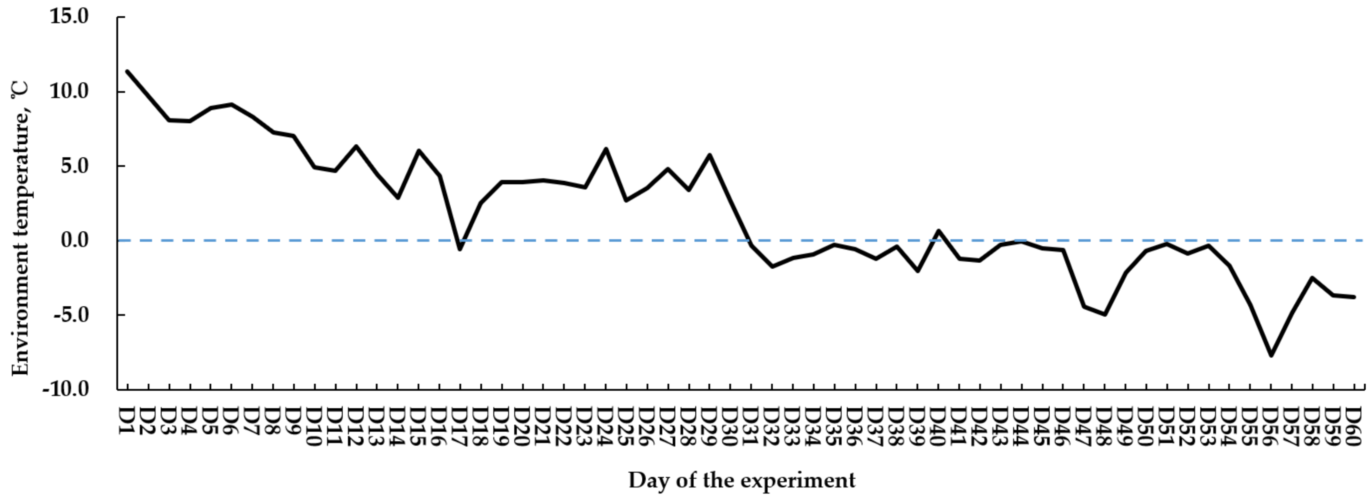

Figure S1. The average daily environment temperature during the experiment (℃).

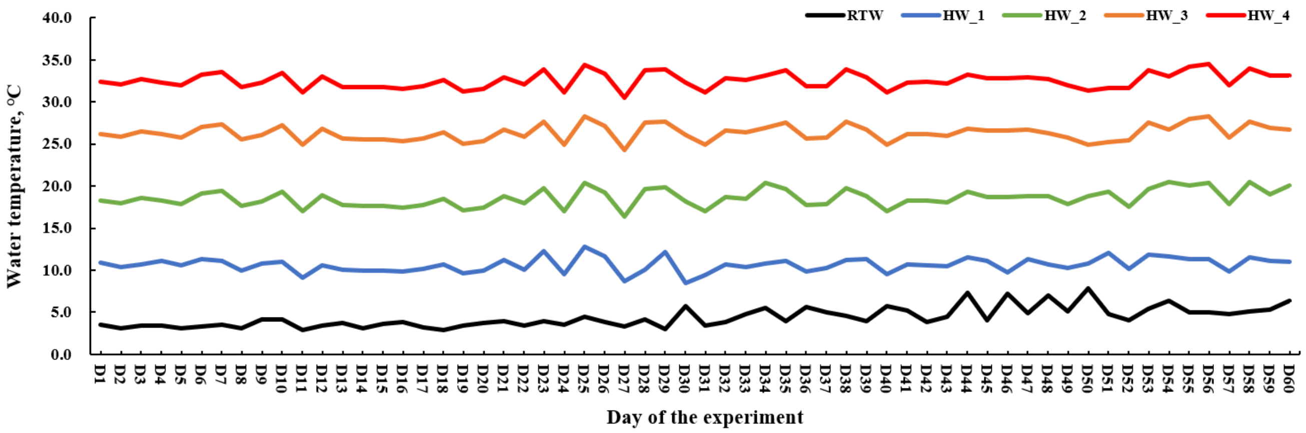

Figure S2. The average daily drinking water temperature of difference treatments during the experiment (℃).

RTW, HW_1, HW_2, HW_3 and HW_4 mean drinking room temperature water at 4.39 ± 2.546℃, 10.63 ± 1.290℃, 18.6 ± 1.52℃, 26.32 ± 1.702℃, and 32.54 ± 2.624℃ respectively.

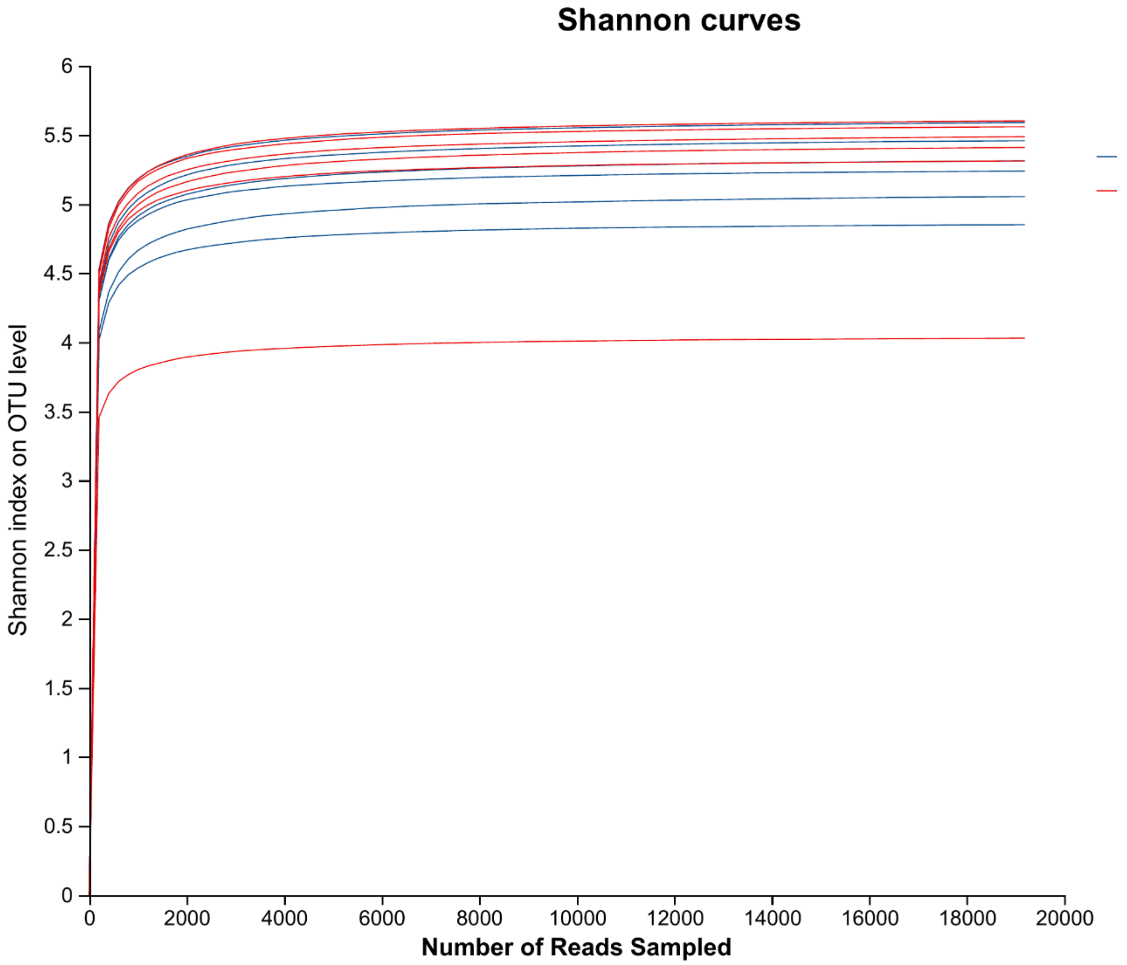

Figure S3. The rarefaction curves of remen liquid at OTU level of fatting cattle drinking heated water or room temperature water. All samples indicated that the diversity indices increased sharply and eventually flatten,suggesting that the sequencing data were substantial enough for reflecting the majority of microbial diversity information in the samples. RTW and HW_2 mean drinking room temperature water at 4.39 ± 2.546℃ and heated water at 18.6 ± 1.52℃, respectively.
